# Multivalvular infective endocarditis with Proteus mirabilis

**DOI:** 10.1016/j.idcr.2022.e01429

**Published:** 2022-01-28

**Authors:** Amber Bux, Ahmad Mustafa, Muhammad Niazi, Umesh Manchandani, Neville Mobarakai, James Lafferty, Vincent DeChavez

**Affiliations:** a Department of Internal Medicine, Staten Island University Hospital, Staten Island, NY, USA; b Department of Infectious Disease, Staten Island University Hospital, Staten Island, NY, USA; c Department of Cardiology, Staten Island University Hospital, Staten Island, NY, USA

**Keywords:** Infective endocarditis, Proteus, Multivalvular

## Abstract

Proteus species belong to the Enterobacteriaceae family and are gram-negative-rods, commonly known to cause urinary tract infections and asymptomatic bacteriuria in elderly patients with risk factors such as diabetes mellitus and urinary catheterization. However, Proteus species are rarely known to cause infective endocarditis. We present a case of an 85-year-old female who presented due to decreased responsiveness with urine and blood cultures growing *Proteus mirabilis*. While she was being treated for her urinary tract infection, her echocardiogram showed vegetation on the left coronary cusp of the aortic valve and left pulmonic valve leaflet. Uncontrolled tachyarrhythmias and new-onset atrial fibrillation complicated the hospital course. Later, she became bradycardic during the hospital stay, and all rate-control medications were held. Unfortunately, she went into cardiac arrest and spontaneous circulation could not be established with resuscitation attempts and she expired. To our knowledge, this is a rare case of native valve infective endocarditis secondary to *Proteus mirabilis*, leading to uncontrolled tachyarrhythmias and death

## Introduction

Proteus species are motile, lactose-negative, urease-producing, Gram-negative bacilli that are part of the Enterobacteriaceae family. Proteus species are well known to be associated with urinary tract infections (UTIs), both in patients without risk factors and in patients with indwelling catheters or abnormal urinary anatomy. Due to its flagella and production of adhesins, *Proteus mirabilis* can form biofilms leading to colonization in the setting of foreign materials such as urinary catheters [1]. Due to these virulence factors they can lead to complicated bloodstream infections and Infective endocarditis (IE) in rare circumstance. Furthermore, due to its rarity, IE caused by gram negative bacteria raises management challenges due to lack of adequate cases and defined treatment guidelines [2]. Treatment guidelines vary with gram negative endocarditis, but bactericidal and synergic agents are commonly seen and preferred over 6 weeks. We present a case of multivalvular native valve IE due to *Proteus mirabilis* bacteremia.

## Case report

85-year-old female with history of dementia, diabetes mellitus type II, chronic kidney disease stage III, congestive heart failure, hypertension, and seizure was sent in from the nursing home for evaluation of hypoxia and decreased responsiveness. She was hypoxic with an oxygen saturation of 80% on room air which improved with 3 liters/minute nasal cannula. She was also febrile with a temperature of 101.3 F and tachypneic with a respiratory rate of 22 breaths per minute.

Physical examination was significant for lethargy and reduced breath sounds and rhonchi in the bilateral lung bases. Chest x-ray showed bilateral basal opacities. Computerized Tomography (CT) angiogram of the chest showed small to moderate bilateral pleural effusions with overlying compressive atelectasis and secretions in the trachea, right mainstem bronchus, and occlusion of peripheral bilateral lower lobe bronchi suspicious for aspiration. Urinalysis was positive for bacteria and leukocyte esterase and negative for nitrite. A urine culture and two sets of blood cultures were obtained on admission. Given her septic presentation, she was empirically started on ampicillin/sulbactam and vancomycin for aspiration pneumonia due to concerning findings on a CT scan.

**Fig. 1:**
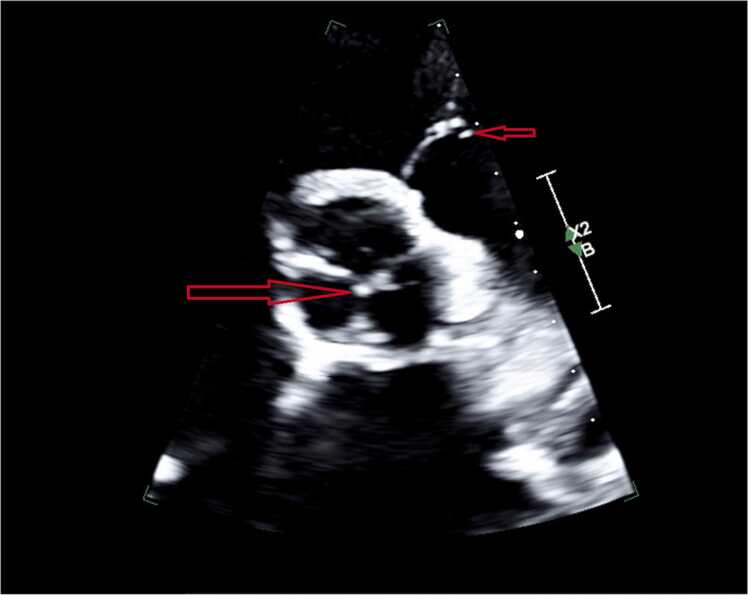
Bottom arrow (bigger) showing vegetation on left coronary cusp of the aortic valve. Top arrow (smaller) showing echogenic mobile density on the left pulmonic valve leaflet.

**Fig. 2:**
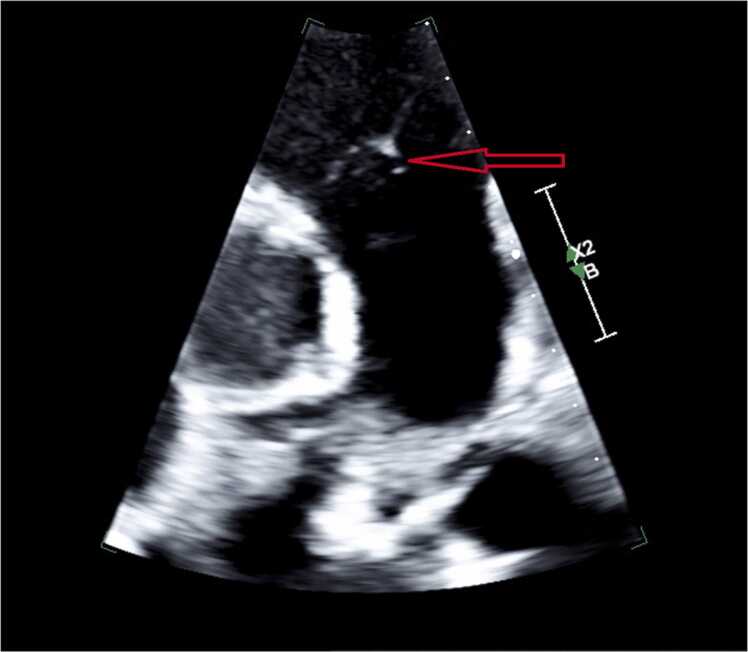
Arrow showing echogenic mobile density on the left pulmonic valve leaflet

**Fig. 3:**
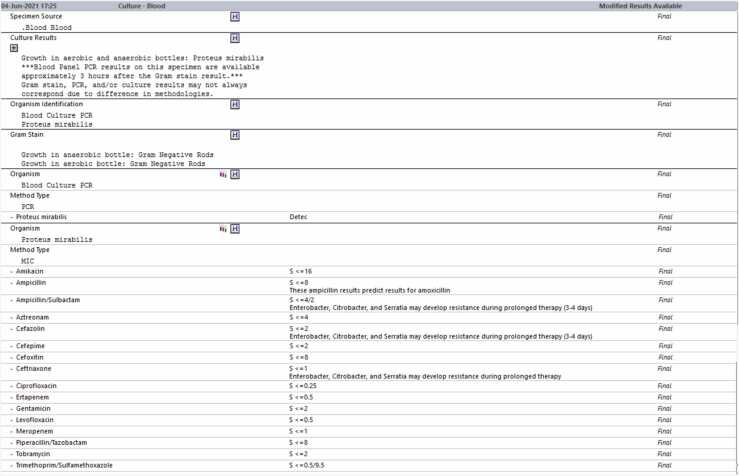
Blood cultures with sensitivities.

Blood cultures drawn on admission were positive for *Proteus mirabilis.* Repeat blood cultures were drawn 24 h later, and remained positive for the growth of *Proteus mirabilis.* A UTI was suspected as the source of the bacteremia, and she was taken off vancomycin and ampicillin/sulbactam and started on meropenem. Transthoracic echocardiogram (TTE) demonstrated a possible mass on the left coronary cusp of the aortic valve and echogenic mobile density on the left pulmonic valve leaflet consistent with vegetation (Fig. 1, Fig. 2). Her antibiotic regimen was switched from Meropenem to Cefepime and was later deescalated to Ceftriaxone based on culture sensitivities (Fig. 3). Subsequent blood cultures were negative, and she was started on 6 weeks of antibiotics course with intravenous Ceftriaxone.

The hospital course was complicated by tachycardia with heart rate around 150 beats per minute (bpm), and an electrocardiogram revealed new-onset atrial fibrillation with a rapid ventricular response. She was initially treated with metoprolol tartrate, but the heart rate remained uncontrolled, so amiodarone was added.

She continued to have tachyarrhythmia episodes that were uncontrolled despite multiple medications and intravenous fluids. She was later found bradycardic with heart rate around 30 bpm . All medications were withheld, however she remained bradycardiac, ultimately leading to cardiac arrest. Resuscitation efforts were performed as per Advanced Cardiovascular Life Support (ACLS) guidelines but were unsuccessful in achieving return of spontaneous circulation and the patient expired.

## Discussion

IE is commonly seen in gram-positive bacteremia due to the formation of biofilms. However, gram-negative bacteria may be involved in some cases of IE, specifically Proteus species. Proteus spp, especially *Proteus mirabilis*, is commonly associated with UTIs. Due to its ability to produce adhesins, it can form biofilms very quickly and lead to bacteremia [2]. Proteus species account for only a small percentage of cases of gram-negative endocarditis, with *Escherichia coli* and *Pseudomonas aeruginosa* being the most common. According to a recent systematic review, only 16 cases of infective endocarditis caused by Proteus species have been reported. *Proteus mirabilis* was identified pathogen in 14 out of 16 cases [3]. Involvement of the aortic valve was the primary site of infection in five out of the fifteen patients, whereas four patients had mitral valve infection. A prosthetic valve was present in 25% of patients and posed a higher risk of infection when compared to a native valve. One patient with a prosthetic valve developed an aortic ring abscess and aortoatrial fistula leading to atrioventricular dissociation and complete heart block. Therefore, the overall mortality rate associated with gram-negative endocarditis is about 50% despite adequate antibiotic therapy and requires early surgical intervention [4].

Proteus species are not considered highly resistant to antibiotics, however, there is mention of almost 33% resistance to co-trimoxazole and quinolones [1]. Aminoglycosides, cephalosporins, and aminopenicillins are among the treatment options considered for IE secondary to Proteus spp. According to American Heart Association guidelines, a synergistic combination of penicillin or cephalosporin and aminoglycoside is recommended; however, no clinical trials or cohort studies support this regimen [5], [6].

Lately there has been a lot of debate about early surgical intervention versus antimicrobial therapy for the treatment. Clinicians have been exploring the option of offering early surgical intervention in patients with severe IE. Conventionally, surgery has been only indicated in severe cases, that is after completion of antibiotic therapy, but the high rate of mortality and complications required a change of treatment approach. The mortality during hospitalization and the first year were recorded as high as 60% and 75%, respectively [7]. American Association for Thoracic Surgery in 2016 concluded that surgical management should be considered in complicated infective endocarditis, characterized by large vegetations (greater than 10 mm in size), recurrent embolization events, persistent sepsis on appropriate medical treatment, acute heart failure, valvular dysfunction, and paravalvular abscesses [6], [8].

The case presented is unique because the patient had multivalvular endocarditis of native valves. Multivalvular endocarditis is rare, but when it does occur, it usually affects the aortic or mitral valves and is caused by *Staphylococcus aureus* or *Streptococcus viridans*. It is also associated with a higher mortality rate and is more common in individuals with congestive heart failure [9]. In our case, an echocardiogram demonstrated a possible mass on the left coronary cusp of the aortic valve and an echogenic mobile density on the left pulmonic valve leaflet consistent with vegetation. The patient was started on the appropriate antimicrobial therapy, but surgical intervention was not possible due to subsequent complications and rapid deterioration. Surgical intervention was assumed to play a major role in the few individuals who survived proteus endocarditis and only few cases have been reported in which the patient survived without surgical intervention. Aggressive measures should be taken for management, and antibiotics should be continued for at least 6 weeks following the clearance of blood culture. In addition, the high likelihood of complications from IE such as tachyarrhythmias and heart failure should be treated as early as possible to minimize mortality.

## Consent

Written informed consent was obtained from the patient for publication of this case report and accompanying images. A copy of the written consent is available for review by the Editor-in-Chief of this journal on request.

## Funding

This research did not receive any specific grant from funding agencies in the public, commercial, or not-for-profit sectors.

## CRediT authorship contribution statement

**Amber Bux:** Conceptualization, Resources, Writing – original draft. Ahmad Mustafa: Conceptualization, Resources, Writing – review & editing. **Muhammad Niazi:** Resources, Writing – review & editing. Umesh Manchandani: Writing – review & editing. **Neville Mobarakai:** Conceptualization, Resources, Supervision. **James Lafferty:** Resources, Supervision. **Vincent DeChavez:** Conceptualization, Resources, Writing – review & editing, Supervision.
